# A Rare Case of Occult Advanced Uterine Adenocarcinoma With a Neoplastic Enterouterine Fistula on Presentation

**DOI:** 10.7759/cureus.27271

**Published:** 2022-07-26

**Authors:** Rina Ghorpade, Vyshnavi Iswaravaka, Nitant Parekh, Fiona Tissavirasingham

**Affiliations:** 1 Internal Medicine, Canton Medical Education Foundation, Northeast Ohio Medical University (NEOMED), Canton, USA; 2 Geriatrics, University of Maryland, Baltimore, Baltimore, USA

**Keywords:** mri, ct abdomen and pelvis, enterouterine fistula, endometrial cancer, advanced uterine cancer

## Abstract

Enterouterine fistula is a rare complication of endometrial carcinoma, typically associated with a clinical presentation of malodorous vaginal discharge. We present a case of occult metastatic endometrial cancer with an initial presentation of embolic stroke, further complicated by an incidental finding of enterouterine fistula on imaging. This case uniquely describes a patient with no symptoms suggestive of uterine malignancy or enterouterine fistula, highlighting a rare presentation of this occult malignancy and further emphasizing the importance of preventative screening.

## Introduction

Uterine cancer is the most common gynecological malignancy in developed countries and the second most common in developing countries after cervical cancer. It is the most prevalent gynecological malignancy in the United States, and it is estimated that 65,950 people will be diagnosed this year, accounting for 12,550 deaths [1]. Uterine cancer usually presents abnormal vaginal bleeding, abdominopelvic discomfort, abnormal vaginal discharge, or bowel/bladder dysfunction [2]. However, the presentation of advanced uterine cancer with enterouterine fistula is sporadic, and only about 20 cases have been reported in the literature thus far [3]. The infrequent involvement of the uterus in the formation of this fistula is related to the thick muscularity of this organ, resisting infiltration of its cavity by malignancy or inflammation. Therefore, reporting a varied presentation of this rare complication is essential to develop an understanding and awareness of enterouterine fistulas in the setting of endometrial carcinoma.

## Case presentation

A 63-year-old Caucasian female with a past medical history of anxiety, osteoarthritis status post right-sided knee replacement, and left hip replacement presented to the emergency department with generalized weakness, right upper extremity weakness, and speech difficulty lasting for 30 minutes. On examination, the patient did not have any neurological signs. Complete blood count showed leukocytosis with a white blood cell count of 21,000/microliter, microcytic anemia with hemoglobin of 7.5 mg/dl, and thrombocytosis with a platelet count of 483/microliter. Chest X-ray and computed tomography (CT) of the head without contrast were negative for acute abnormalities. CT angiogram of the head/neck showed right vertebral artery occlusion and focal irregular eccentric soft tissue atheroma of the posterior aortic arch concerning the embolic disease. Neurology was consulted. She did not receive a tissue plasminogen activator (tPA), as she was out of the therapeutic benefit window. Magnetic resonance imaging (MRI) of the brain showed the distal right vertebral artery occlusion and acute non-hemorrhagic multifocal small infarcts involving vascular territories concerning for stroke, likely embolic in nature. The patient was initially started on aspirin and atorvastatin for secondary stroke prevention.

Interestingly, the patient was undergoing outpatient workup with hematology/oncology for leukocytosis and microcytic anemia. Upon detailed history, the patient mentioned feeling fatigued for the last four to six months and had lost almost 30 pounds. The patient denied any symptoms of vaginal bleeding, vaginal discharge, abdominal pain, or bloating. Additionally, she never underwent any cancer screening. A CT of the abdomen and pelvis and CT of the thorax were ordered, given her hematologic dyscrasias and symptoms.

**Figure 1:**
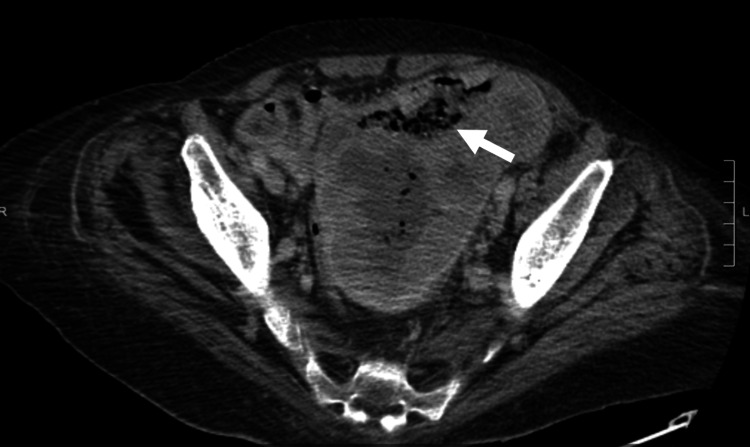
CT scan of the abdomen and pelvis showing complex air bubbles in the uterine cavity (white arrow) suggesting a fistulous tract between the uterine cavity and the small intestine.

The leukocytosis continued to worsen to 31,000/microliter. Upon reviewing external records from hematology/oncology, it was revealed that the patient had been worked up for hematological malignancy. However, the workup for hematological malignancy was negative. CT of the abdomen and pelvis showed a significant uterine/endometrial malignancy with small bowel fistula (Figure 1) and metastatic right common iliac and para-aortic adenopathy.

Subsequently, gynecologic/oncology was consulted. A physical exam showed normal external genitalia. The cervix was nulliparous and smooth. On palpation, a huge mass was found beginning in the lower uterine segment and filling the posterior cul-de-sac. The mass was completely fixed in the pelvis and was palpable up to the level of the umbilicus, extending out to the sidewalls. No palpable groin lymph nodes were noted. Additionally, the rectal exam was normal.

The patient underwent an endometrial biopsy of the mass and interventional radiology (IR)-guided drain placement for a complex fluid collection. The endometrial biopsy showed moderately to poorly differentiated adenocarcinoma with extensive necrosis with moderately differentiated glandular proliferation and other areas of undifferentiated malignant sheets of cells.

Unfortunately, the patient’s hospital course was complicated by septic shock secondary to small bowel perforation and *Klebsiella pneumoniae* bacteremia. She was placed on multiple antibiotics and supportive treatment. Gynecology/oncology did not think that the patient would be a good surgical candidate given the metastatic nature of the disease and other comorbidities. The palliative care team was consulted, and the patient’s code status was changed to comfort care. Eventually, the patient passed away within three weeks of her hospital admission.

## Discussion

The presentation of advanced uterine cancer can be nonspecific and includes bleeding from the vagina, rectum, or bladder if the disease is locoregional. It can also present as abdominal/pelvic pain, weight loss, bone pain, or symptoms of anemia, including shortness of breath and fatigue [4]. Concerning our patient, the only symptoms she presented with that sparked any suspicion for occult malignancy were weight loss and fatigue, making the diagnosis of endometrial carcinoma complicated by an enterouterine fistula an unexpected finding.

Enterouterine fistulas are communicating tracts between the intestine and the uterus. They may result from uterine trauma, advanced colorectal cancers or pelvic tumors, post-surgical complications of gynecological or surgical procedures, and inflammatory bowel diseases. The enterouterine fistula was first reported in 1909, and three main etiologies were postulated, including injury to the uterus, rupture of a pyometra into the bowel, and uterine or colorectal malignancies [5]. The typical clinical presentation of patients with enterouterine fistula includes malodorous vaginal discharge that can be fecal or purulent [6]. Interestingly, our patient did not have these symptoms.

Our patient was diagnosed with an enterouterine fistula based on initial CT imaging; however, many other diagnostic modalities are available, including MRI, hysteroscopy, and surgical exploration. CT findings of enterouterine fistulas include air bubbles in the uterine cavity or colonic wall; however, the sensitivity of CT scan to detect a fistulous tract is low. MRI is a more accurate diagnostic modality compared to CT imaging. Interestingly, a case reported in 2004 described an enterouterine fistula diagnosed using sonohysterography by observing the flow of ultrasound contrast medium between the uterine cavity and the sigmoid colon [7]. The definitive diagnosis, however, can only be established with hysteroscopy and surgical exploration [8]. Thus, while CT and MRI are essential for precise preoperative evaluation and planning, hysteroscopy allows for direct visualization of the fistulous tract.

The treatment of an enterouterine fistula in most cases is surgical management. Surgical management depends on the patient’s risks, comorbidity, and stage of the disease. En bloc resection is necessary in cases of enterouterine fistula caused by malignancy [6]. But in cases where the etiology of enterouterine fistula is not malignancy, colon resection and/or drainage of an abscess can be sufficient. Our patient had extensive metastatic disease with multiple comorbidities, including sepsis, embolic stroke, bowel perforation, and frailty. Therefore, a palliative approach was used, and she underwent IR-guided abscess drainage.

## Conclusions

This case highlights that the presentation of endometrial cancer with concurrent enterouterine fistulas can be subtle and present without obvious symptoms, especially in those who have not undergone age-appropriate screening. This case also raises the question if patients with an established diagnosis of endometrial cancer should receive periodic abdomen/pelvis imaging. Further documentation of such cases will continue to add to the growing body of knowledge guiding screening and management.
